# Association between Secondhand Smoke in Hospitality Venues and Urinary 4-(methylnitrosamino)-1-(3-pyridyl)-1-butanol Concentrations in Non-Smoking Staff

**DOI:** 10.3390/ijerph13111101

**Published:** 2016-11-08

**Authors:** Jeonghoon Kim, Kiyoung Lee, Ho-Jang Kwon, Do Hoon Lee, KyooSang Kim

**Affiliations:** 1 Department of Environmental Health Research, Seoul Medical Center, 156 Sinnae-ro, Jungnang-gu, Seoul 131-795, Korea; xellos88@naver.com; 2 Department of Environmental Health, Graduate School of Public Health, Seoul National University, 1 Gwanak-ro, Gwanak-gu, Seoul 151-742, Korea; cleanair@snu.ac.kr; 3 Institute of Health and Environment, Graduate School of Public Health, Seoul National University, 1 Gwanak-ro, Gwanak-gu, Seoul 151-742, Korea; 4 Department of Preventive Medicine, Dankook University College of Medicine, 119 Dandae-ro, Dongnam-gu, Cheonan 448-701, Korea; hojang@dankook.ac.kr; 5 National Cancer Center, 323 Ilsan-ro, Ilsandong-gu, Goyang-si 410-769, Korea; dhlee@ncc.re.kr

**Keywords:** cotinine, hospitality venue, NNAL, PM_2.5_, secondhand smoke

## Abstract

The purpose of this study was to determine the relationship between urinary cotinine and total 4-(methylnitrosamino)-1-(3-pyridyl)-1-butanol (NNAL) concentrations in non-smoking staff and the indoor levels of fine particles (PM_2.5_) in hospitality venues that allow smoking, with respect to demographic and indoor environmental factors. We evaluated 62 hospitality venues that allowed smoking in Seoul, Korea. A real-time aerosol monitor was used to measure indoor PM_2.5_ concentrations. Field technicians recorded indoor environmental characteristics. One non-smoking staff member in each hospitality venue was tested for urinary cotinine and total NNAL concentrations. Demographic characteristics were obtained from self-reported staff questionnaires. Natural-log (ln)-transformed PM_2.5_ concentrations were significantly correlated with the ln-transformed cotinine (*r* = 0.31) and the total NNAL concentrations (*r* = 0.32). In multivariable regression analysis, the urinary cotinine concentrations of the staff members were significantly correlated with indoor PM_2.5_ concentrations; those with the highest concentrations were more likely to be women or staff members that worked in venues with a volume <375 m^3^. Total NNAL concentrations were significantly correlated only with indoor PM_2.5_ concentrations. Indoor PM_2.5_ may be used as an indicator for urinary cotinine and total NNAL concentrations in non-smoking staff members in hospitality venues that allow smoking.

## 1. Introduction

Secondhand smoke (SHS) contains more than 7000 chemicals, including more than 69 known carcinogens [1]. Exposure to SHS is associated with cardiovascular disease, respiratory disease, and lung cancer [2,3,4,5]. Based on data from 192 countries, SHS exposure caused 603,000 premature deaths in 2004 [6]. In the United States, SHS accounted for the death of more than 41,000 adults and approximately 900 infants in 2006 [7].

Based on increased evidence of adverse health effects due to SHS exposure, many countries have implemented smoke-free regulations in public places, including hospitality venues. Airborne markers for SHS exposure, such as particulate matter smaller than 2.5 μm (PM_2.5_), have been used to demonstrate SHS exposure in hospitality venues [8,9]. Previous studies have reported that implementation of smoke-free regulations significantly reduced indoor PM_2.5_ concentrations in hospitality venues [8,9].

Although indoor PM_2.5_ concentrations can be measured easily using a real-time monitor that provides comparatively accurate readings, PM_2.5_ is not a tobacco-specific marker [10]. One method of quantifying human SHS exposure is to measure levels of tobacco-specific biomarkers, such as cotinine. Cotinine is the major metabolite of nicotine in body fluids with average half-life of 16 h [11]. SHS contains tobacco-specific nitrosamine-(methylnitrosamino)-1-(3-pyridyl)-1-butanone (NNK) [12]. NNK has been classified as a Group 1 carcinogen by the International Agency for Research on Cancer (IARC) [13]. Total 4-(methylnitrosamino)-1-(3-pyridyl)-1-butanol (NNAL) which consists of NNAL and its glucuromides (NNAL-Glucs), is metabolized from NNK with a half-life of up to three weeks in urine [14]. NNAL is considered as havingsimilar adverse health effects as NNK [15]. Since the half-life of NNAL is longer than that of cotinine, NNAL can represent cumulative SHS exposure over a longer period of time [16]. Urinary cotinine and total NNAL concentrations have been used to quantify the SHS exposure of non-smoking staff members in hospitality venues [17].

Since most studies use either airborne markers or biomarkers to measure SHS exposure in hospitality venues, it is necessary to assess how these markers are related. Several studies have assessed the relationship between indoor PM_2.5_ concentrations and urinary cotinine concentrations in hospitality venues [18,19]; these studies showed a significant correlation between indoor PM_2.5_ concentrations and urinary cotinine concentrations in non-smoking staff. To our knowledge, only one study has assessed the relationships between indoor PM_2.5_ concentrations and total NNAL concentrations in non-smoking staff working in semi-open air cafés [20]. That study reported that indoor PM_2.5_ concentrations were correlated with urinary total NNAL concentrations. However, associations between urinary cotinine and total NNAL concentrations in non-smoking staff and indoor PM_2.5_ concentrations that take into account characteristics, such as indoor environmental factors, were not well characterized. The purpose of this study is to assess the relationship between urinary cotinine and total NNAL concentrations in non-smoking staff members based on indoor PM_2.5_ concentrations, as well as demographic and indoor environmental factors, including indoor volume, ventilation status, and smoking status.

## 2. Materials and Methods

### 2.1. Study Design

This study used data from a previous study that assessed air quality and biomarker levels in non-smoking staff members at restaurants and bars before and after the implementation of Korean smoke-free regulations [21]. The data in this study were, therefore, collected before the implementation of smoke-free regulations in restaurants and bars (April and June 2013). The research team and the Korean Health Center called restaurants and bars randomly to determine whether they allowed smoking. When indoor smoking was allowed, restaurants and bars were selected if their owners or managers were interested in participating in the study. After this first contact, the research team visited the venues to explain the study’s objectives and procedures. Staff members who had never been smokers, former smokers who had quit at least three months before the study, and staff that worked in the main hall were selected. Urinary samples and demographic questionnaires were collected from the staff members. Indoor PM_2.5_ concentrations in 71 hospitality venues were measured as close as possible to ±1 week from the collection of urinary samples. Although 95 non-smoking staff members in the 71 hospitality venues had their biomarker levels measured [21], we selected staff members who had complete urinary cotinine and total NNAL concentration data, including indoor PM_2.5_ data for their venues. If there was more than one staff member in a venue that fit the criteria, we selected the staff member whose birthday was earliest in the year. In total, 62 non-smoking staff members from individual venues were selected. All staff members participated voluntarily and provided written informed consent. The ethics committee at Seoul Medical Center reviewed and approved all procedures prior to the survey (IRB No. 2013-006).

### 2.2. Airborne Marker Levels for SHS

PM_2.5_ concentrations were measured using a real-time photometric aerosol monitor (SidePakTM, Model AM 510, TSI Inc., Shoreview, MN, USA). The monitor was installed with a 2.5-μm impactor and the airflow rate was set at 1.7 L/min. The monitor was calibrated to zero using a high-efficiency particulate absorption filter prior to measurement and was set to a logging interval of 1 min. The measured data from the monitor were adjusted by a factor of 0.295, which was obtained from a previous experiment using a SidePak monitor co-located with a gravimetric measurement [22].

The researchers visited restaurants between 18:00 and 20:00 and bars between 20:00 and 24:00 on weekdays. The monitor was hidden in a small bag, with its inlet protruding out of the bag. Indoor PM_2.5_ concentrations were measured for 30 min (*n* = 30) and outdoor PM_2.5_ concentrations were measured for 5 min before and after the indoor measurement (*n* = 10). An average PM_2.5_ concentration value was determined by averaging 1-min data points from each indoor and outdoor venue. In the venues, the monitor was placed on a table or a seat away from windows, doors, kitchen areas, and direct emission sources (e.g., cigarette smokers). The researchers recorded information on the type of hospitality venue, size, window status, and observation of smoking and counted the numbers of customers and vents in the venue. The number of customers was counted every 5 min and averaged to estimate the average number of customers over the course of the visit. Customer and vent densities were calculated as the number of customers and vents per 100 m^2^ of indoor area. When outdoor PM_2.5_ concentrations were measured, the monitor was placed away from direct emission sources, such as smokers and outside vents.

### 2.3. Biological Marker Levels for SHS

The researchers collected spot-urine samples and self-reported staff demographic questionnaires in the venues during staff breaks or setup time. The urine samples were frozen at −70 °C for storage until analysis. The frozen samples were sent to the Center for Clinical Services, National Cancer Center, and the analysis was conducted in a blinded manner to the type of hospitality venue and the smoking history of the participants.

The urinary cotinine concentrations were measured by liquid chromatography-tandem mass spectrometry (LC-MS/MS) using a method modified from a previous study [23]. An Agilent 1100 series liquid chromatography unit (Agilent Technologies, Santa Clara, CA, USA) was used and an API 4000 machine (AB Sciex, Framingham, MA, USA) equipped with an atmospheric pressure chemical ionization interface was used as a tandem mass spectrometer. Urinary total NNAL concentrations were measured by LC-MS/MS using a method modified from a previous study [24]. The limit of quantification (LOQ) was 2 ng/mL for urinary cotinine and 0.25 pg/mL for total NNAL concentration. Half of the LOQ was assigned when the urinary cotinine and total NNAL concentrations were below the LOQ. Urinary creatinine concentrations were measured using a colorimetric approach (Toshiba 2090 FR; Toshiba, Tokyo, Japan). Participants whose urinary cotinine levels exceeded 100 ng/mL were excluded because they were suspected to be smokers [25].

### 2.4. Demographic Information

The study used face-to-face questionnaires to obtain the participants’ demographic information [21]. Demographic factors such as sex, age, employment position, weekly working hours, and whether the participant lived with a smoker were used as variables.

### 2.5. Statistical Analysis

The normality of the data was assessed using the Shapiro-Wilk test. Since indoor PM_2.5_, urinary cotinine, and total NNAL concentrations were not normally distributed, they were log-transformed for statistical analysis. We present the measured data as geometric means (GMs) and geometric standard deviations (GSDs). Pearson’s correlation test was used to assess the relationship between indoor PM_2.5_, urinary cotinine, and total NNAL concentrations. We describe indoor PM_2.5_ according to indoor environmental characteristics, such as the type of hospitality venue (restaurant or bar), volume (<375 m^3^ or ≥375 m^3^), customer density (<10 customers/100 m^2^ or ≥10 customers/100 m^2^), vent density (<7 vents/100 m^2^ or ≥7 vents/100 m^2^), window status (closed or open), and smoking observed (yes or no). The volume (ranging from 90 to 1680 m^3^), customer density (ranging from 1 to 53 customers/100 m^2^), and vent density (ranging from 0 to 40 vents/100 m^2^) were divided into two groups based on their median values. Spearman’s correlation test was used to assess the associations between indoor PM_2.5_ concentrations and the type of hospitality venue, customer density, and observations of smoking.

We described urinary cotinine and total NNAL concentrations according to demographic characteristics, such as sex (women and men), age (<45 years, 45–54 years, and ≥55 years), employment position (owner or manager and temporary or permanent staff), working hours (<70 h/wk and ≥70 h/wk), whether the participant lived with a smoker at home (yes or no), and the indoor environmental factors. The number of working hours (ranging from 22 to 91 h/wk) was divided into two groups based on median values. Indoor PM_2.5_, urinary cotinine, and total NNAL concentrations were compared using Student’s *t-*test for two groups of variables and a general linear model analysis for more than two groups of variables.

Using variables with *p*-values < 0.1 identified in the univariable analysis, a multivariable regression analysis was conducted to identify the relationships between urinary cotinine and total NNAL concentrations and indoor PM_2.5_ concentrations including other variables. A stepwise method was used in the regression models. In all of our analyses, a *p*-value of 0.05 was deemed significant. SAS 9.3 (SAS Institute Inc., Cary, NC, USA) was used for statistical analysis.

## 3. Results

The GMs of the indoor and outdoor PM_2.5_ concentrations in the 62 hospitality venues were 65.3 μg/m^3^ (GSD = 2.2) and 33.8 μg/m^3^ (GSD = 1.6), respectively. The indoor and outdoor PM_2.5_ concentrations differed significantly (*p* < 0.001). In the univariable analysis, several indoor environmental factors were significantly associated with indoor PM_2.5_ concentrations (Table 1), which were significantly higher in bars than in restaurants (*p* < 0.001). Indoor PM_2.5_ concentrations were significantly higher in venues with a customer density ≥10 customers/100 m^2^ than in those with <10 customers/100 m^2^ (*p* < 0.001), and were significantly higher in venues where smoking was observed than in venues where smoking was not observed (*p* < 0.001). However, indoor PM_2.5_ concentrations were not associated with the volume, vent density, or window status of the venues.

Indoor PM_2.5_ concentrations were significantly correlated with the type of hospitality venue (*r* = 0.56; *p* < 0.001), customer density (*r* = 0.47; *p* < 0.001), and observation of smoking (*r* = 0.52; *p* < 0.001) (Table 2). The type of hospitality venue was significantly correlated with customer density (*r* = 0.39; *p* = 0.002) and observation of smoking (*r* = 0.60; *p* < 0.001). Customer density was significantly correlated with observations of smoking (*r* = 0.39; *p* = 0.002).

Of the 62 urinary samples collected from the staff members, urinary cotinine levels were above the LOQ in 19 samples (31%) and total NNAL values were above the LOQ in 62 samples (100%). The GMs of urinary cotinine and total NNAL concentration of the 62 staff members were 1.8 ng/mg Cr (GSD = 2.8) and 7.3 pg/mg Cr (GSD = 2.5), respectively. Natural log (ln)-transformed indoor PM_2.5_ concentrations were significantly correlated with ln-transformed urinary cotinine concentrations (*r* = 0.31; *p* = 0.013) and with ln-transformed total NNAL concentrations (*r* = 0.32; *p* = 0.011) (Table 3). Pearson’s correlation coefficients between ln-transformed urinary cotinine concentrations were significantly correlated with ln-transformed urinary total NNAL concentrations (*r* = 0.39; *p* = 0.002).

Several demographic and indoor environmental factors were significantly associated with urinary cotinine and total NNAL concentrations (Table 4). Urinary cotinine concentrations were significantly higher in female staff members than in male staff members (*p* < 0.001), and were significantly higher among staff members who worked in bars than in those who worked in restaurants (*p* = 0.040). They were also significantly higher in staff members who worked in venues with volumes <375 m^3^ than in those who worked in venues with volumes ≥375 m^3^ (*p* = 0.005). The cotinine concentrations of staff members who worked in venues with customer densities of ≥10 customers/100 m^2^ were significantly higher than those of staff members who worked in venues with customer densities of <10 customers/100 m^2^ (*p* = 0.049). Cotinine concentrations were significantly higher in staff members who worked in venues with closed windows than in those who worked in venues with open widows (*p* = 0.021). Cotinine concentrations were significantly higher in staff members who worked in venues where smoking was observed than in those who worked in venues where smoking was not observed (*p* < 0.001). However, the cotinine concentrations of staff members were not significantly associated with age, living with a smoker at home, employment position, working hours, or vent density in the venues.

The total NNAL concentrations of staff members who worked in bars were significantly higher than those of staff members who worked in restaurants (*p* = 0.002). The total NNAL concentrations were significantly higher in staff members who worked in venues with volumes <375 m^3^ versus ≥375 m^3^ (*p* = 0.046). The total NNAL concentrations were significantly higher in staff members who worked in venues where smoking was observed than in those who worked in venues where smoking was not observed. However, the NNAL concentrations of staff members were not significantly associated with sex, age, living with a smoker, employment position, working hours, customer or vent densities, or window status.

Multivariable regression analyses of ln-transformed urinary cotinine and total NNAL concentrations were performed using ln-transformed indoor PM_2.5_ concentrations and demographic and indoor environmental factors with *p* < 0.1, as identified in the univariable analyses. Since indoor PM_2.5_ concentrations were significantly associated with the type of hospitality venue, customer density, and smoking observation status, these variables were not included in the multivariable regression analysis.

Urinary cotinine concentrations were significantly associated with indoor PM_2.5_ concentrations, sex, and volume, and were marginally associated with window status (*R*^2^ = 0.39; Table 5). The urinary cotinine concentrations of staff members were positively associated with indoor PM_2.5_ concentrations (*p* = 0.003), and were significantly higher in female staff members than in male staff members (*p* = 0.001). The urinary cotinine concentrations of staff members who worked in venues with indoor volumes <375 m^3^ were significantly higher than those of staff members who worked in venues with indoor volumes ≥375 m^3^ (*p* = 0.038), and were marginally higher in staff members who worked in venues with closed windows versus open windows (*p* = 0.065).

Total NNAL concentrations were significantly associated with indoor PM_2.5_ concentrations and were marginally associated with volume (*R*^2^ = 0.19). The total NNAL concentrations of staff members were positively associated with indoor PM_2.5_ concentrations (*p* = 0.012). The total NNAL concentrations were marginally higher in staff members who worked in venues with indoor volumes <375 m^3^ than in those who worked in venues with indoor volumes ≥375 m^3^ (*p* = 0.090). However, window status was not associated with the total NNAL concentrations.

## 4. Discussion

The GM of indoor PM_2.5_ concentrations in 62 hospitality venues was 65.3 μg/m^3^, 1.6-fold higher than the National Ambient Air Quality Standard (NAAQS) for the daily PM_2.5_ concentration of 35 μg/m^3^. The levels were above the NAAQS in 72% of the hospitality venues. Of the hospitality venues, indoor PM_2.5_ concentrations were above the NAAQS in 51% of restaurants and 100% of bars. High levels of indoor PM_2.5_ concentrations have been reported in hospitality venues not covered by smoke-free regulations. In Scotland, the average indoor PM_2.5_ concentration in 41 bars before smoke-free regulations were imposed was 246 μg/m^3^ [26]. In Georgetown, Kentucky, the average indoor PM_2.5_ concentration in nine hospitality venues and one bingo hall, before the introduction of smoke-free regulations, was measured at 84 μg/m^3^ [9].

Several indoor environmental factors were associated with indoor PM_2.5_ concentrations in hospitality venues. Indoor PM_2.5_ concentrations were significantly higher in bars, those with customer densities ≥10 customers/100 m^2^, and those in which smoking was observed. Smoking is a significant emission source, and can contribute 90%–95% of respirable particles in hospitality venues [27]. Higher levels of indoor PM_2.5_ concentrations in bars than in restaurants may be explained by the presence of smokers. In this study, smoking was observed in 14% of restaurants and 74% of bars during the measurement of indoor PM_2.5_ concentrations. Other possible sources in hospitality venues are human activity, cooking, and outdoor air pollution. Human activity, cooking, and smoking may be associated with customer density. When more customers are present in a hospitality venue, these activities are likely to increase and, in turn, indoor PM_2.5_ concentrations may be associated with customer density. A previous study reported differences between indoor and outdoor PM_2.5_ concentrations in 109 bars were significantly correlated with the number of patrons (Spearman’s *r* = 0.197, *p* < 0.05) [28].

Our study found significant correlations between indoor PM_2.5_ concentrations and the urinary cotinine and total NNAL concentrations of staff members in hospitality venues. Although the correlation coefficients between indoor PM_2.5_ concentrations and urinary cotinine and total NNAL concentrations were similar, higher correlation coefficients have been found between urinary cotinine and total NNAL concentrations. Previous studies in semi-open-air cafés have found that indoor PM_2.5_ concentrations were significantly correlated with urinary cotinine concentrations in non-smoking staff (Spearman’s *r* = 0.914, *n* = 49, *p* < 0.001) [18] and that indoor PM_2.5_ concentrations were significantly correlated with urinary total NNAL concentrations (Spearman’s *r* = 0.378, *n* = 49, *p* < 0.01) in non-smoking staff [20].

The correlation coefficients between indoor PM_2.5_ concentrations and the urinary cotinine concentrations of non-smoking staff members were lower in our study than in a previous study conducted in semi-open-air cafés [18]. The correlation coefficients between indoor PM_2.5_ concentrations and urinary total NNAL concentrations were slightly lower than in another previous study conducted in semi-open-air cafés [20]. The low correlation coefficients between indoor PM_2.5_ and urinary cotinine and total NNAL concentrations in our study may be because most urine samples were not collected after work on the day of the measurement of indoor PM_2.5_ concentrations. Conversely, previous studies collected urine samples from non-smoking staff after their shift at the end of the day, on the same day that the PM_2.5_ concentrations were measured [18,20]. In our study, the average difference in the number of days between the collection of urine samples and the measurement of indoor PM_2.5_ concentrations was −2.4 ± 5.7 days.

In our multivariable analysis, several factors, including indoor PM_2.5_ concentrations, were significantly associated with urinary cotinine and total NNAL concentrations in hospitality venue staff members. Sex was the only significant factor among the demographic factors for urinary cotinine concentrations. The urinary cotinine concentrations of female staff members were higher than those of male staff members. However, total NNAL concentrations were not associated with any other demographic factor evaluated. Previous studies have reported that demographic factors, such as sex and age, are not associated with urinary cotinine [18] or total NNAL concentrations in non-smoking staff [20]. Possible reasons for higher cotinine levels in female staff members include that women are less prone to report that they smoke because they smoke intermittently. These women may have been included in the analysis, although their cotinine levels were not as high as current smokers. Another possible reason may have been that women worked more hours than did men over the week preceding the study. The average working hours over the previous seven days was 67 ± 15 h for female staff members (*n* = 44) and 59 ± 17 h for male staff members (*n* = 18). Longer working hours for female staff members may contribute to greater SHS exposure, and this may have resulted in higher urinary cotinine concentrations than those found in the male staff members.

Urinary cotinine and total NNAL concentrations were not consistent by sex. Urinary cotinine and total NNAL concentrations may have yielded different results, because cotinine, a major metabolite of nicotine, is a more specific and sensitive biomarker for SHS exposure than is total NNAL. Cotinine has an average half-life of 16 h [11] and is discharged from the human body within 3–4 days after SHS exposure [29]. However, NNAL reflects SHS exposure for longer than does cotinine, with a half-life of up to three weeks [11]. Intermittent smoking and the longer working hours of female staff members may have resulted in higher urinary cotinine levels. However, urinary total NNAL concentrations in men and women may reflect overall SHS exposure over several weeks due to NNAL’s longer half-life.

Indoor volume was significantly associated with urinary cotinine concentrations in hospitality venue staff members. Indoor volume was marginally associated with urinary total NNAL concentrations. The urinary cotinine and total NNAL concentrations in the hospitality venues with indoor volumes <375 m^3^ were 2.2- and 1.6-fold higher, respectively, than those in venues with indoor volumes ≥375 m^3^. The findings of this study indicate that staff members who work in hospitality venues with relatively small volumes may be at risk of higher SHS exposure than those who work in hospitality venues with relatively large volumes.

Window status was not significantly associated with urinary cotinine or total NNAL concentrations in our multivariable analysis. In addition to window status, vent density was not significantly associated with urinary cotinine or total NNAL concentrations in our univariable analysis. The findings of our study indicate that natural or mechanical ventilation does not protect staff from SHS exposure in hospitality venues.

Hospitality venues without indoor smoking showed significantly lower levels of urinary cotinine and total NNAL concentrations. The finding suggested that implementation of smoke-free regulations in hospitality venues could protect staff from SHS exposure. A previous study reported that urinary cotinine concentrations of 40 non-smoking staff member in bars decreased from 35.9 to <5 ng/mL in 6–10 weeks after the implementation of smoke-free regulations in Michigan, USA [17]. These staff members also showed a 60% reduction of NNAL concentrations after the implementation of smoke-free regulations.

Key strengths of our study include the use of one non-specific airborne marker (indoor PM_2.5_) [10] and two specific biomarkers (urinary cotinine and total NNAL) [11] to characterize the relationship of the urinary cotinine and total NNAL concentrations in non-smoking staff members with indoor PM_2.5_ concentrations in hospitality venues, and another strength is the inclusion of demographic and indoor environmental factors. Total NNAL concentrations, metabolites of carcinogenic NNK, were used to estimate staff exposure to SHS in hospitality venues.

However, our study has several limitations. Restaurants and bars were not selected randomly. The selected hospitality venues and their staff may not be representative of the more general population. We chose different times to visit for the types of venues (i.e., between 18:00 and 20:00 for restaurants and between 20:00 and 24:00 for bars) because the time might be the most crowded time for each type of venue. Indoor PM_2.5_ concentrations were measured for 30 min at each venue. However, the short measurement time might not cause under or over estimation of indoor air quality. Another limitation is that we did not collect staff urine samples on the same day that PM_2.5_ measurements were taken. Some urine samples were collected several days before or after measurement of the indoor PM_2.5_ concentrations. Therefore, the urinary cotinine and total NNAL values of nonsmoking staff in the hospitality venues may not directly reflect SHS exposure on the day of the indoor PM_2.5_ measurements. The study did not account for non-combustible tobacco products in non-smoking staff member because they were not commonly used.

## 5. Conclusions

In total, 62 hospitality venues that allowed smoking and their non-smoking staff members were evaluated to determine the relationships between urinary cotinine and total NNAL concentrations and indoor PM_2.5_ concentrations with respect to demographics and indoor environmental factors. Indoor PM_2.5_ concentrations were significantly correlated with urinary cotinine and total NNAL concentrations in staff members in the hospitality venues. Including indoor PM_2.5_, we found the urinary cotinine concentrations in the staff members to be significantly associated with sex and indoor room volume. Total NNAL concentrations in staff members were significantly associated only with indoor PM_2.5_ concentrations. Our findings suggest that indoor PM_2.5_ concentrations may be useful indicators for urinary cotinine and total NNAL concentrations in non-smoking staff members in hospitality venues that allow smoking.

## Figures and Tables

**Table 1:** Univariable analysis of indoor PM_2.5_ concentrations (μg/m^3^) by indoor environmental factors in hospitality venues (*n* = 62).

Variable		*n*	GM (GSD)	*p-*Value
Type of Hospitality Venue	Restaurant	35	44.7 (2.0)	<0.001
	Bar	27	106.9 (2.0)	-
Volume (m^3^)	<375	31	69.4 (2.4)	0.554
	≥375	31	61.4 (2.1)	-
Customer Density (customers/100 m^2^)	<10	32	45.2 (1.9)	<0.001
	≥10	30	96.7 (2.2)	-
Vent Density (vents/100 m^2^)	<7	32	67.6 (2.1)	0.730
	≥7	30	62.9 (2.4)	-
Window	Closed	36	65.6 (2.3)	0.963
	Open	26	64.9 (2.2)	-
Smoking Observed	No	37	46.2 (1.9)	<0.001
	Yes	25	108.9 (2.2)	-

PM_2.5_: particulate matter smaller than 2.5 μm; GM: geometric mean; GSD: geometric standard deviation.

**Table 2:** Spearman correlation coefficients among indoor PM_2.5_ concentration, type of hospitality venue, customer density, and observation of smoking in the venues (*n* = 62).

Variable	Indoor PM_2.5_ Concentration	Type of Hospitality Venue ^a^	Customer Density ^b^	Smoking Observed ^c^
Indoor PM_2.5_ Concentration	1.00	-	-	-
Type of hospitality Venue	0.56 **	1.00	-	-
Customer Density	0.47 **	0.39 *	1.00	-
Smoking Observed	0.52 **	0.60 **	0.39 *	1.00

^a^ Restaurant vs. bar; ^b^ <10 vs. ≥10 customers/100 m^2^; ^c^ No vs. Yes; * *p* < 0.01; ** *p* < 0.001.

**Table 3:** Pearson’s correlation coefficients among natural log (ln)-transformed PM_2.5_, urinary cotinine, and total NNAL concentrations in staff members of hospitality venues (*n* = 62).

Substance	Ln-Transformed Indoor PM_2.5_	Ln-Transformed Urinary Cotinine	Ln-Transformed Urinary Total NNAL
Ln-Transformed Indoor PM_2.5_	1.00	-	-
Ln-Transformed Urinary Cotinine	0.31 *	1.00	-
Ln-Transformed Urinary Total NNAL	0.32 *	0.39 **	1.00

NNAL: 4-(methylnitrosamino)-1-(3-pyridyl)-1-butanol; * *p* < 0.05; ** *p* < 0.01.

**Table 4:** Univariable analysis of urinary cotinine and total NNAL concentrations in staff members by demographic and indoor environmental factors in hospitality venues (*n* = 62).

Variable		*n*	Cotinine (ng/mg Cr)		Total NNAL (pg/mg Cr)	
			GM (GSD)	*p-*Value	GM (GSD)	*p-*Value
**Demographic Factors**		-	-	-	-	-
Sex	Women	44	2.3 (2.7)	<0.001	7.4 (2.5)	0.836
	Men	18	0.9 (2.0)	-	7.0 (2.5)	-
Age (Years)	<45	17	1.7 (3.1)	0.947	8.0 (2.4)	0.346
	45–54	23	1.8 (2.8)	-	5.9 (2.1)	-
	≥55	22	1.9 (2.6)	-	8.6 (2.9)	-
Living with a Smoker at Home	No	37	1.7 (2.5)	0.344	7.4 (2.3)	0.467
	Yes	25	2.1 (2.7)	-	8.1 (2.5)	-
Employment Position	Owner/Manager	37	1.7 (2.5)	0.577	7.4 (2.3)	0.881
	Temporary/Permanent Staff	25	2.0 (3.2)	-	7.2 (2.7)	-
Working Hours (h/wk)	<70	28	1.8 (2.8)	0.963	8.8 (2.4)	0.145
	≥70	34	1.8 (2.8)	-	6.3 (2.5)	-
**Indoor Environmental Factors**		-	-	-	-	-
Type of Hospitality Venue	Restaurant	35	1.4 (2.1)	0.040	5.4 (2.2)	0.002
	Bar	27	2.4 (3.5)	-	10.9 (2.4)	-
Volume (m^3^)	<375	31	2.6 (3.1)	0.005	9.2 (2.6)	0.046
	≥375	31	1.2 (2.2)	-	5.8 (2.3)	-
Customer Density (customers/100 m^2^)	<10	32	1.4 (2.4)	0.049	6.5 (2.3)	0.317
	≥10	30	2.3 (3.0)	-	8.2 (2.6)	-
Vent Density (vents/100 m^2^)	<7	32	1.7 (2.8)	0.571	8.2 (2.5)	0.334
	≥7	30	1.9 (2.8)	-	6.5 (2.4)	-
Window	Closed	36	2.3 (2.9)	0.021	8.7 (2.4)	0.079
	Open	26	1.3 (2.3)	-	5.8 (2.5)	-
Smoking Observed	No	37	1.3 (1.9)	<0.001	5.3 (2.0)	<0.001
	Yes	25	3.0 (3.5)	-	11.8 (2.6)	-

GM: geometric mean; GSD: geometric standard deviations; Cr: creatinine.

**Table 5:** Multivariable analysis of natural log (ln)-transformed urinary cotinine (ng/mg Cr) and total NNAL concentrations (pg/mg Cr) with ln-transformed PM_2.5_ concentrations (μg/m^3^) and other variables (*n* = 62).

Substance	Variable	Coefficient	Standard Error	*p*-Value
Ln-Transformed Urinary Cotinine (*R*^2^ = 0.39)	Intercept	−0.49	0.58	0.402
	Ln-Transformed Indoor PM_2.5_	0.41	0.13	0.003
	Sex	-	-	-
	Men (Reference: Women)	−0.81	0.24	0.001
	Volume	-	-	-
	≥375 m^3^ (Reference: <375 m^3^)	−0.46	0.22	0.038
	Window	-	-	-
	Open (Reference: Closed)	−0.41	0.22	0.065
Ln-Transformed Urinary Total NNAL (*R*^2^ = 0.19)	Intercept	0.89	0.59	0.133
	Ln-Transformed Indoor PM_2.5_	0.34	0.13	0.012
	Volume	-	-	-
	≥375 m^3^ (Reference: <375 m^3^)	−0.37	0.21	0.090
	Window	-	-	-
	Open (Reference: Closed)	−0.36	0.22	0.104
